# A need for speed: Objectively identifying full-body kinematic and neuromuscular features associated with faster sprint velocities

**DOI:** 10.3389/fspor.2022.1094163

**Published:** 2023-02-03

**Authors:** Chris L. Vellucci, Shawn M. Beaudette

**Affiliations:** Spine Biomechanics and Neuromuscular Control Lab, Faculty of Applied Health Sciences, Brock University, St. Catharines, ON, Canada

**Keywords:** biomechanics, machine learning, sprinting, coordination, sports performance, objective movement assessment

## Abstract

Sprinting is multifactorial and dependent on a variety of kinematic, kinetic, and neuromuscular features. A key objective in sprinting is covering a set amount of distance in the shortest amount of time. To achieve this, sprinters are required to coordinate their entire body to achieve a fast sprint velocity. This suggests that a whole-body kinematic and neuromuscular coordinative strategy exists which is associated with improved sprint performance. The purpose of this study was to leverage inertial measurement units (IMUs) and wireless surface electromyography (sEMG) to find coordinative strategies associated with peak over-ground sprint velocity using machine learning. We recruited 40 healthy university age sprint-based athletes from a variety of athletic backgrounds. IMU and sEMG data were used as inputs into a principal components analysis (PCA) to observe major modes of variation (i.e., PC scores). PC scores were then used as inputs into a stepwise multivariate linear regression model to derive associations of each mode of variation with peak sprint velocity. Both the kinematic (*R*^2 ^= 0.795) and sEMG data (*R*^2 ^= 0.586) produced significant multivariate linear regression models. The PCs that were selected as inputs into the multivariate linear regression model were reconstructed using multi-component reconstruction to produce a representation of the whole-body movement pattern and changes in the sEMG waveform associated with faster sprint velocities. The findings of this work suggest that distinct features are associated with faster sprint velocity. These include the timing of the contralateral arm and leg swing, stance leg kinematics, dynamic trunk extension at toe-off, asymmetry between the right and left swing side leg and a phase shift feature of the posterior chain musculature. These results demonstrate the utility of data-driven frameworks in identifying different coordinative features that are associated with a movement outcome. Using our framework, coaches and biomechanists can make decisions based on objective movement information, which can ultimately improve an athlete's performance.

## Introduction

Sprint performance is reliant on a variety of kinematic, kinetic, and neuromuscular features which require an athlete to coordinate their upper and lower body to achieve a faster sprint velocity. This suggests that whole-body kinematic and neuromuscular coordinative strategies may exist which are associated with improved sprint performance. Despite this, many studies have assessed specific sub-regions of the body and have selected discrete parameters *a-priori* for statistical analysis. For example, many studies compare differences in muscle activation or changes in range of motion of a joint at a specific time point such as toe-off or touchdown (1–5). Such studies disregard aspects of whole body coordination which suggest that intricate multi-segment coordination is required to complete a motor task (6). Further, these approaches have led to researchers traditionally focusing on the biomechanics of the lower extremity when assessing sprint performance, resulting in a reduced emphasis on the mechanics of the upper extremities and trunk. However, as sprinting is a complex whole-body movement, it is unlikely that the lower extremity biomechanics alone are associated with faster sprint velocities. Thus, there is a need for a comprehensive and holistic understanding of the multi-segment whole-body dynamics associated with faster sprint velocities.

At its simplest, sprint velocity is the by-product of the interaction between stride length and stride rate (7). Stride length and stride rate are mutually dependent variables with an inverse relationship. To accomplish a faster sprint velocity, one must either rely on an increase in stride length or stride rate. These features have been reported to be influenced by a variety of different factors. For instance, stride length appears to be positively influenced by explosive strength, muscle mass, lower extremity length, biological sex, ground reaction force, ground contact duration, and dynamic flexibility of the hips (7–11). Comparatively, stride rate appears to be influenced by rate of force development which can be affected by motor neuron excitability, inter- and intramuscular coordination, fatigue, horizontal velocity of the COM during stance, leg angle touch down, leg angle at take-off, and leg length (7, 8).

To facilitate proper multi-segment coordination, sprinting requires the proper sequencing and timing of various muscles to effectively optimize sprint technique. During sprinting the CNS coordinates the activation and relaxation of various muscles in a rhythmic nature. This requires the use of a pre-set motor program which is refined through the integration of sensory information from systems such as the visual, proprioceptive, and vestibular systems (12). It's previously been shown that sprinters undergo a dynamic control strategy that shifts from ankle dominant to hip dominant strategy when velocities exceed 7.0 m/s. This demonstrates that intricate changes occur in the lower extremity which are associated with faster sprint velocities. What remains unknown is how the musculature of the trunk, specifically those that attach to the thoracolumbar fascia, display timing and magnitude differences as a function of sprint velocity. This is particularly relevant, as a strong theoretical foundation exists that suggests these muscles play an important role in the pendulum like action of the contralateral arm and leg swing during locomotion and may be particularly important during faster sprint velocities (13–15).

To optimize an individual's sprint performance a coach must manipulate an individual's whole-body coordinative strategy to improve sprint velocity. This requires the coach to identify key movement features associated with improved sprint performance using their coaching eye, which lacks the objectivity and sensitivity to truly optimize an individual's health and performance. Recently, the objective quantification of movement technique has grown in popularity. This is largely due to the pressure on the biomechanical community to create an objective framework that can be used to help provide objective insights into how to optimize an individual's movement technique to improve a performance outcome (16). A PCA-based framework has previously been used to create a discriminative model to quantify the effect age, body mass index, biological sex and emotion has on human walking. Further, this framework has since been adapted to model differences in ski technique and classify athletes as novice or advanced (17, 18). One of the main benefits of PCA in biomechanics is its ability to provide easily interpretable models using single component reconstruction (SCR) (19). Using SCR, a full-body avatar can be reconstructed to provide athletes and coaches with an easy-to-use technique to communicate key technical differences between individuals and/or to provide longitudinal feedback to an athlete or coach.

### Purpose

The purpose of this research is to leverage wearable sensor technology and data-driven tools to objectively assess the kinematic and neuromuscular determinants of over-ground sprint velocity through the analysis of a large dataset of university-aged sprinters. This hypothesis-generating study will serve as the foundation for future work related to the development of customizable data-driven sprint coaching tools to discriminate along biomechanically relevant axes of ability performance ability and rehabilitation status.

### Hypothesis statement

It was hypothesized that several distinct kinematic and neuromuscular features would be present as significant contributors to the prediction of sprint velocity. These expected discriminative features included differences in the spatiotemporal coordinative strategy of the thorax and pelvis, and the timing and activation of the muscles attached to the thoracolumbar fascia.

## Materials and methods

### Participants

41 participants (27 male, 14 females; mean ± standard deviation age: 21.8 ± 3.2 years; height: 176.8 ± 8.4 cm) from a variety of team sports and track and field events were recruited for this study. Demographic information is presented in Table 1. One participant was removed from the sample due to issues with data quality. Participants were required to be recreationally active at least twice a week in a sprint-based sport. Participants must not have reported any neurological, cardiovascular, or muscular disorders that may impact their sprint performance and have no known allergies to rubbing alcohol or adhesives. The current protocol was approved by the institutional research ethics board in accordance with the Canadian Tri-Council Policy Statement (TCPS 2) on the Ethical Conduct for Research Involving Humans.

**Table 1:** Mean** ±** SEM participant demographics.

Demographic	Sample size	Age	Male	Female	Height
Rugby	6	21 ± 2.5	4	2	178 ± 20.5
Sprinting	4	20 ± 1.8	3	1	175 ± 14.2
Soccer	15	21.9 ± 3.7	10	5	174.6 ± 3.7
Ice Hockey	7	22.3 ± 3.0	3	4	178.0 ± 9.4
Other	8	23 ± 3.0	8	0	180.4 ± 4.1

### Experimental protocol

**Figure 1:**
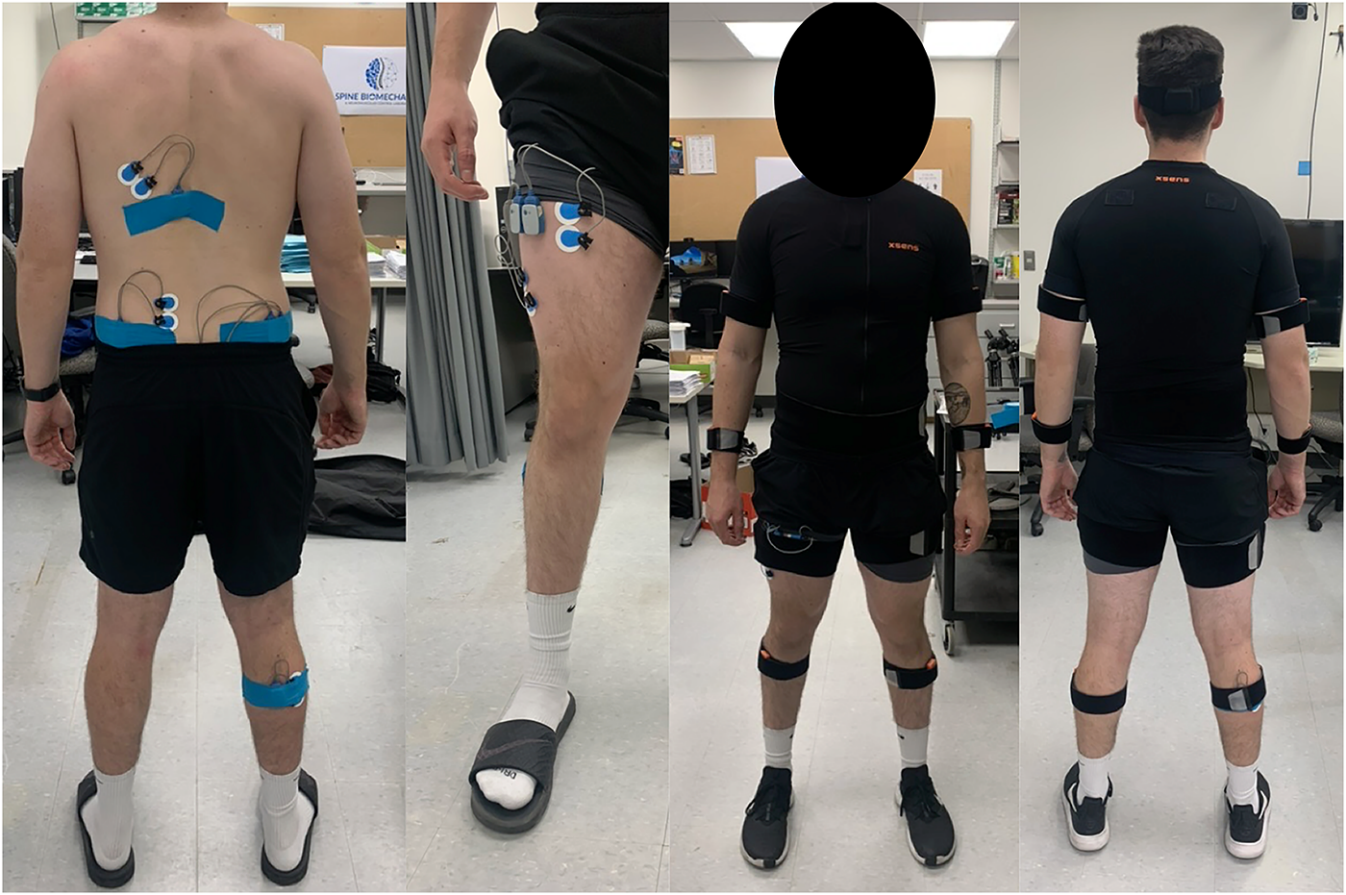
An example of the IMU and sEMG setup on the participant.

Each participant completed a single experimental visit lasting approximately two hours. All participants were instrumented with two types of wearable sensors. Full-body kinematics were collected using a 17-sensor IMU suit, and sEMG were recorded using wireless sensors. IMU sensors (XSens, Awinda) were placed bilaterally on the feet, shank, thigh, upper arm, forearm, hands, and shoulders, with single sensors placed on the sternum, pelvis, and head. All equipment was placed and calibrated as per manufacturer instructions. All kinematic data were acquired at a frequency of 60 Hz. Muscle activation was recorded for nine muscles located from the lower body to upper body. Bipolar surface electrodes and sensors (Noraxon, Ultium) were placed according to SENIAM guidelines on the right lateral gastrocnemius (GAS), right biceps femoris (BF), right gluteus maximus (GMAX), right gluteus medius (GMED), right vastus lateralis (VLO), right rectus femoris (RF), left lumbar erector spinae (LES), left latissimus dorsi (LD), and left external obliques (EO). All sEMG data were acquired at a frequency of 2000Hz. For a visual overview of the experimental setup please refer to Figure 1.

Following instrumentation, the participant underwent a self-directed warm-up for 5 min. Once the participant completed the warmup, they then completed three 60 m over-ground sprints on synthetic track, each separated by a period of at least 5 min of passive rest to avoid any influence of neuromuscular fatigue. During these over-ground sprints, 3D whole-body kinematics and neuromuscular activity were recorded simultaneously. The trial with the fastest peak sprint velocity achieved was selected and used for all further analyses.

### Kinematic data analysis

3D kinematic data were high definition (HD) reprocessed using XSens MVN Analyze software and a 64-marker point-cloud was exported into a .c3d file. The .c3d file was imported into MATLAB (2021b) using Biomechanical Tool Kit 5 (20). The origin of the coordinate system for the imported data was reset so that the x, y, z positions of the right ankle at the beginning of the sprinting trial represented the origin. To correct positional drift during the sprinting trials, PCA was used to re-align the global coordinate system (GCS) of all sprinting trials to ensure that all positional data were aligned across all sprints, and for all participants. Specifically, PCA was used to obtain the three highest 3D components of variation in the XYZ, 64-marker dataset, across all timepoints, for each sprint. Next, 3D rotations were computed between each 3D loading vector and the original global coordinates to derive a 3D rotational offset. Once obtained, this rotational offset was applied to all markers such that the new x-axis corresponded to the axis of progression (i.e., PC1), the new y-axis corresponded to the mediolateral axis (i.e., PC2), and the new z-axis corresponded to the vertical axis (i.e., PC3) for each participant/sprint. This allowed 3D coordinate data for all participants and sprints to be registered to the same 3D coordinate axes prior to further analyses, while also offsetting any effects of IMU sensor drift, or GCS misalignment (during calibration) with respect to the axis of sprint progression. Once all data were realigned, all data were cropped to only include 60 m of sprinting. This was done by determining the frame which the x-position of the T8 marker reached 60 m.

Following 3D point cloud registration, sprint velocity during the 60 m sprint was calculated by first calculating the Euclidean norm of the T8 marker and then calculating its first derivative. The magnitude and point of the maximal velocity was then identified, which was used to inform the selection of the five cycles (about peak velocity) used for our analysis. To facilitate the segmentation of individual cycles, the local minima of the z-position of the right ankle marker was used. After five separate strides were partitioned, they were then each time normalized to 101 frames using a polynomial spline function and subsequently de-biased by subtracting the location of the T12 marker (x, y, z) from all 64 markers in the dataset. This was done to ensure that there was no forward progression of the sprinter, thereby removing any effects of forward progression on the assessment of spatiotemporal coordination.

After stride segmentation, time-normalization, and bias removal, the five strides about maximal velocity were then ensemble averaged, and subsequently scaled by dividing each participants height and reshaped to a 1 × 19,392 (64 markers * 3 axes * 101 data points) vector. The 1 × 19,392 vector for each participant (representing an average stride about peak velocity during the fastest sprinting trial) was then used to construct a PCA matrix, where each row represented a participant, and each column represented the time-varying series of the x, y, and z position of each marker. The result was a 40 × 19,392 data matrix (40 participants * 64 markers * 3 axes * 101 data points) which was used as an input for the PCA (Equation 1).


(1) $$\begin{array}{rl} & [\begin{array}{ccc} M1{\left(x,y,z\right)}_{{\frac{1}{1}}_{-101}} & M2{\left(x,y,z\right)}_{{\frac{1}{1}}_{-101}} & M3{\left(x,y,z\right)}_{{\frac{1}{0}}_{-101}}\\ M1{\left(x,y,z\right)}_{{\frac{2}{1}}_{-101}} & M2{\left(x,y,z\right)}_{{\frac{2}{1}}_{-101}} & M3{\left(x,y,z\right)}_{{\frac{2}{1}}_{-101}}\\ M1{\left(x,y,z\right)}_{{\frac{n}{1}}_{-101}} & M2{\left(x,y,z\right)}_{{\frac{n}{1}}_{-101}} & M3{\left(x,y,z\right)}_{{\frac{n}{1}}_{-101}} \end{array}\\ & \times \begin{array}{ccc} M4{\left(x,y,z\right)}_{{\frac{1}{1}}_{-101}} & \ldots & M64{\left(x,y,z\right)}_{{\frac{1}{1}}_{-101}}\\ M4{\left(x,y,z\right)}_{{\frac{2}{1}}_{-101}} & \ldots & M64{\left(x,y,z\right)}_{{\frac{2}{1}}_{-101}}\\ M4{\left(x,y,z\right)}_{{\frac{n}{1}}_{-101}} & \ldots & M64{\left(x,y,z\right)}_{{\frac{n}{1}}_{-101}} \end{array}] \end{array}$$

**Figure 2:**
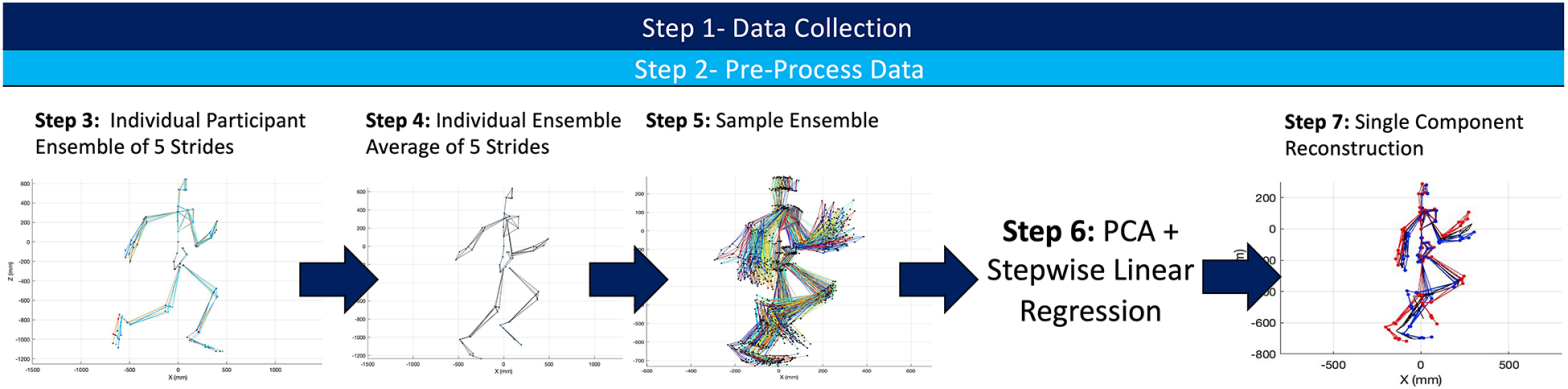
Summary of experimental workflow for both the kinematic and sEMG data set. A similar process was complete for both kinematic and sEMG data to create two different PCA frameworks.

A visual depiction of the analytical steps taken in the processing of any raw kinematic data is depicted in Figure 2.

### sEMG data analysis

Raw sEMG data were imported into MATLAB (2021b). All sEMG data were full wave rectified and low pass filtered using a dual pass 2nd order Butterworth filter with a cut-off of 250 Hz. The filter cut-off was determined using a residual analysis (See Supplementary Figure S1 and Table S1) (21). The filtered data were amplitude normalized by averaging the maximum of the three highest peaks in the first 20 m of the sprint. It has been suggested that this technique may be superior over normalization to maximum voluntary isometric contraction (MVIC) due to the sEMG values being obtained in similar neural condition (i.e., dynamic sprinting) as MVICs fail to reflect the neural drive in dynamic high velocity contractions and can create challenges in interpretation (22). Following amplitude normalization, the analyses of the sEMG signals mirrored the approach taken for the kinematic data. The normalized sEMG data was then partitioned into five cycles and time normalized using a polynomial function to 2000 data points. These cycles were selected about the point of maximal velocity. The data was then reshaped to a 1 × 18,000 vector (9 sEMG channels * 2000 data points). Each participant's vector was then complied into a 40 × 18,000 data matrix which was used as an input for the PCA (Equation 2).


(2) $$\begin{array}{rl} & [\begin{array}{ccc} \mathrm{EMG}1_{{\frac{1}{1}}_{-1000}} & \mathrm{EMG}2_{{\frac{1}{1}}_{-1000}} & \mathrm{EMG}3_{{\frac{1}{0}}_{-1000}}\\ \mathrm{EMG}1_{{\frac{2}{1}}_{-1000}} & \mathrm{EMG}2_{{\frac{2}{1}}_{-1000}} & \mathrm{EMG}3_{{\frac{2}{1}}_{-1000}}\\ \mathrm{EMG}1_{{\frac{n}{1}}_{-1000}} & \mathrm{EMG}2_{{\frac{n}{1}}_{-1000}} & \mathrm{EMG}3_{{\frac{n}{1}}_{-1000}} \end{array}\\ & \begin{array}{ccc} \mathrm{EMG}4_{{\frac{1}{1}}_{-1000}} & \ldots & \mathrm{EMG}9_{{\frac{1}{1}}_{-1000}}\\ \mathrm{EMG}4_{{\frac{2}{1}}_{-1000}} & \ldots & \mathrm{EMG}9_{{\frac{2}{1}}_{-1000}}\\ \mathrm{EMG}4_{{\frac{n}{1}}_{-1000}} & \ldots & \mathrm{EMG}9_{{\frac{n}{1}}_{-1000}} \end{array}] \end{array}$$

### Feature selection and regression analysis

Following the application of the PCA to the kinematic and sEMG data matrices, PCs that explained >95% of the variance in the dataset were retained for further analysis. Simple linear regressions were performed on the PC scores against maximal sprint velocity (m/s). After it was determined that multiple PCs had weak-to-moderate correlations with sprint velocity, a stepwise multivariate linear regression analysis was completed. Dependent variables for the kinematic and sEMG stepwise regression models included age, sex, height, and the retained PCs for each model (i.e., kinematic and sEMG). The stepwise linear regression function used both forward and backward stepwise search mode. The stepwise regression had a tolerance criterion of *p* > 0.10. If the variable added had *p*-values that exceeded the exit tolerance, then the variable with the largest *p*-value was removed. Any PC variables that were determined to be significant contributors to the kinematic and sEMG stepwise linear regression model were subsequently reconstructed using single (i.e., SCR) and multicomponent component reconstruction (MCR). Specifically, SCR and MCR were used to reconstruct an upper and lower limit that provides insight into functional meaning of each PC included within the multivariate linear regression models. SCR provide a functional interpretation of the biomechanical meaning of each PC by representing how each feature is scaled an individual PC (i.e., Equations 3, 4). MCR (i.e., Equations 5, 6) was implemented to understand how all the PCs included in our stepwise potentially interacted in the representation of sprint performance. This provided a more holistic insight into the scaling of features that are represented in the stepwise linear regression models.


(3) $${\hat{x}}_{U}=\;\bar{x}\;\;+\;\;u_{R}\;*\;z_{95}$$

(4) $${\hat{x}}_{L}=\;\bar{x}\;\;+\;\;u_{R}\;*\;z_{05}$$

(5) $${\hat{x}}_{U}=\;\bar{x}\;\;+\;\;u_{1}\;*\;z_{95}+\;u_{2}\;*\;z_{95}+\;u_{3}\;*\;z_{95}+\ldots \;u_{n}\;*\;z_{95}$$

(6) $${\hat{x}}_{L}=\;\bar{x}\;\;+\;\;u_{1}\;*\;z_{05}+\;u_{2}\;*\;z_{05}+\;u_{3}\;*\;z_{05}+\ldots \;u_{n}\;*\;z_{05}$$

## Results

**Figure 3:**
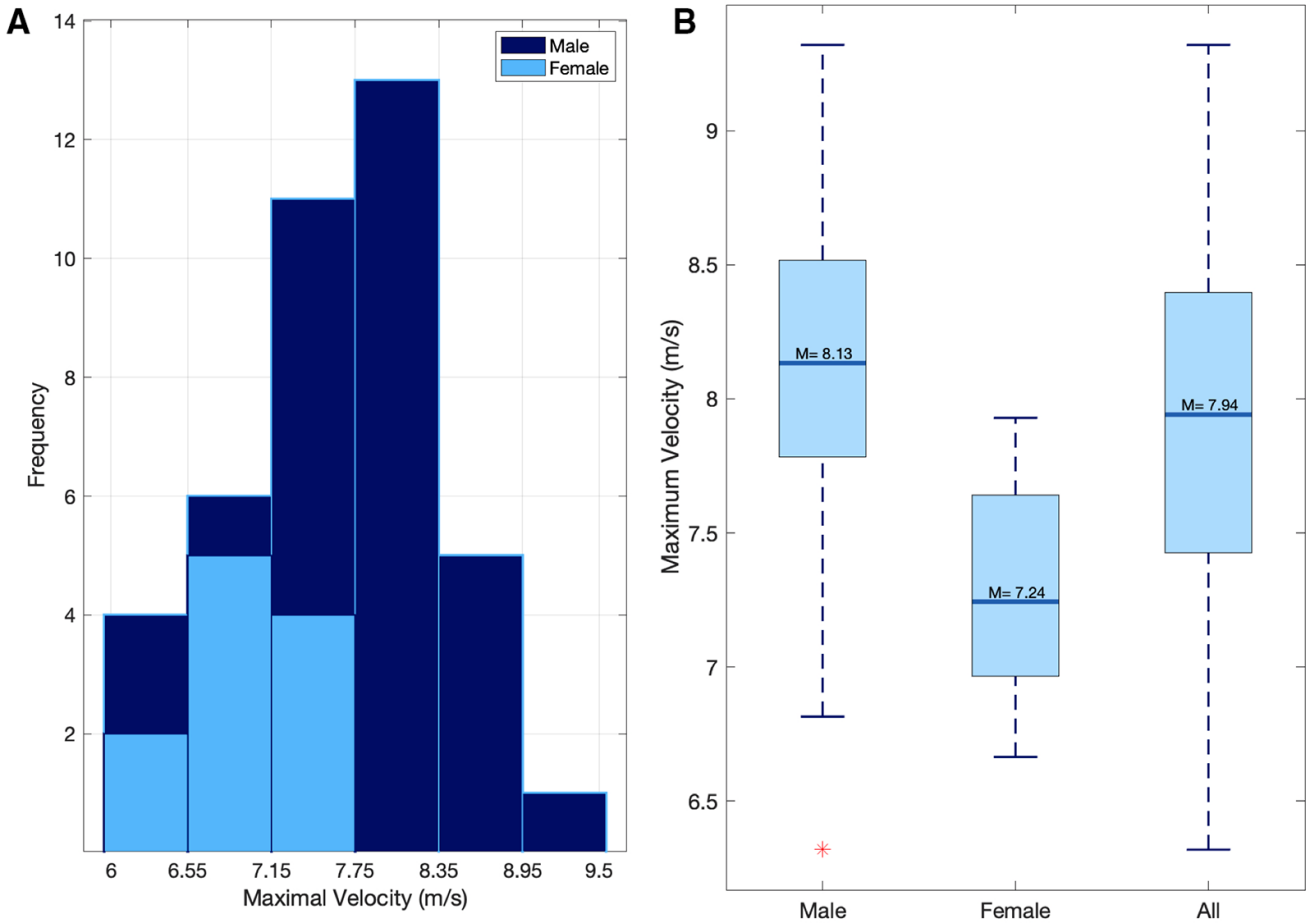
(**A**) the distribution of peak sprint velocities for both males and females. (**B**) The mean sprint velocity for Male, female, and all participants.

**Figure 4:**
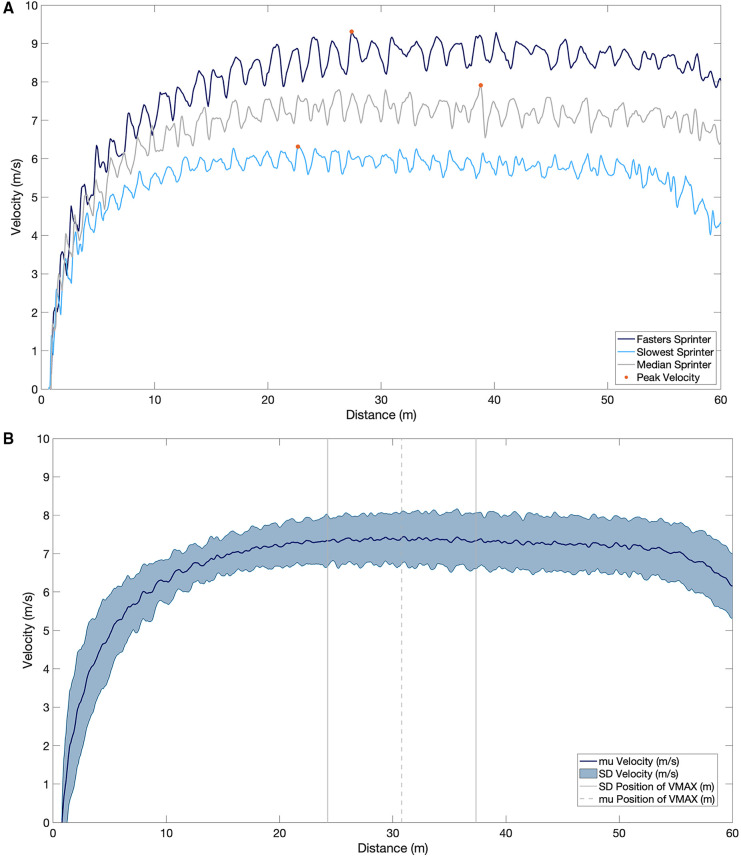
(**A**) the velocity profile of the fastest sprinter (dark blue), slowest sprinter (light blue) and median sprinter (grey) for the entire 60 m. Peak velocity is represented by the orange dot on each velocity profile. (**B**) The mean and standard deviation velocity profile over the 60 m sprint and the mean and standard deviation of the position of the maximal velocity.

The mean peak velocity during the 60 m sprint was 7.94 ± 0.69 m/s (males = 8.13 ± 0.58, females = 7.24 ± 0.56) (Figures 3A,B, 4A,B).

### Principal component analysis and linear regression (kinematics)

**Figure 5:**
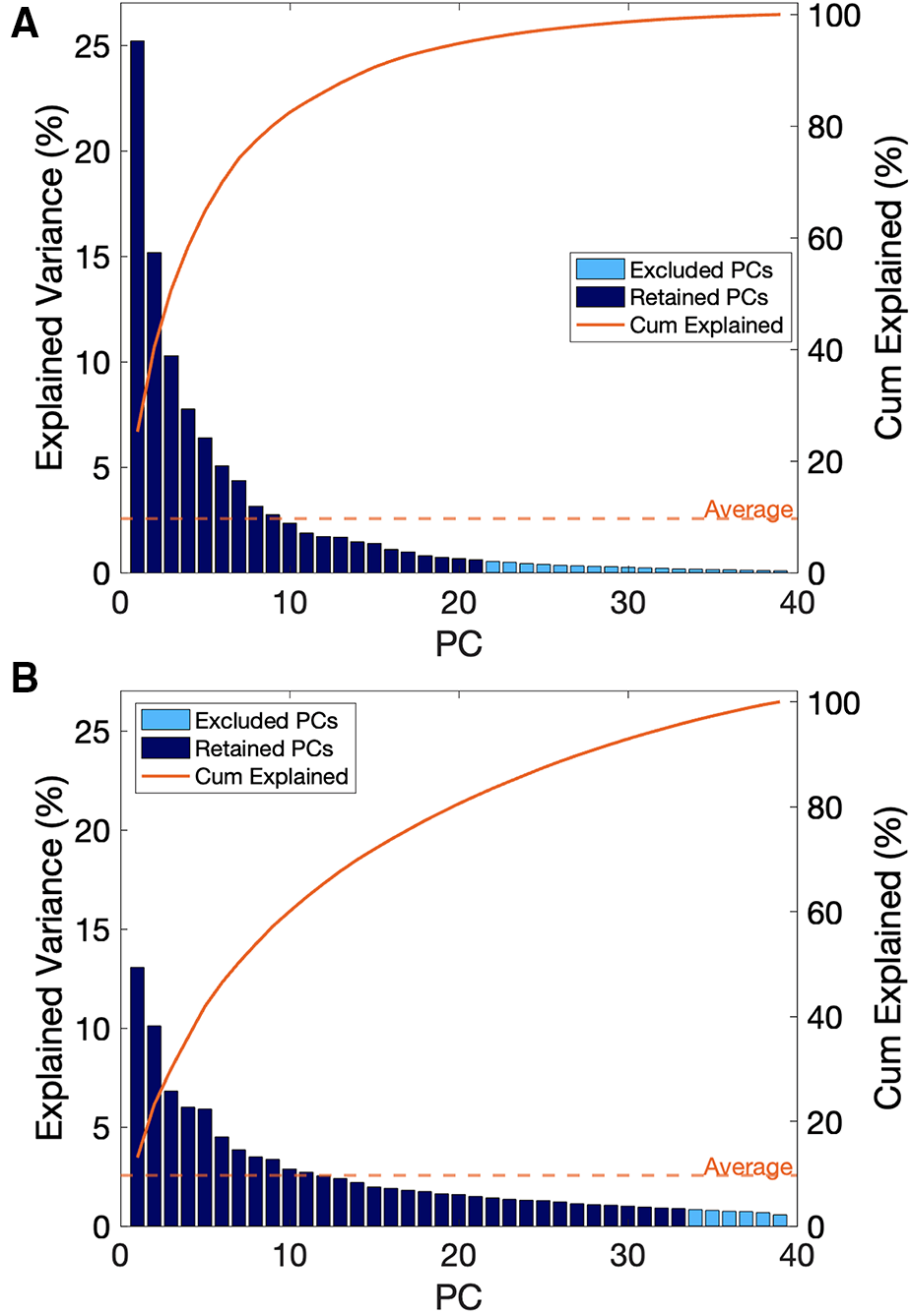
(**A**) scree plot for principal components derived from kinematic data, (**B**) scree plot for principal components derived from sEMG data.

In the assessment of the kinematic data, the first 21 PCs were retained, which explained a cumulative variance of 95.4% in the kinematic dataset (Figure 5A). Stepwise linear regression revealed that PC 1 (*p* = 0.0002), PC 3 (*p* = 0.0119), PC 9 (*p* = 0.0055), PC 11 (*p* = 0. 0066), PC 12 (*p* = 0.0772), PC 13 (*p* = 0.0130), PC 16 (*p* = 0.00004) and biological sex (*p* = 0.0255) were significantly associated with the dependent variable, which was maximal horizontal sprint velocity. The linear regression model displayed a *R*^2^ = 0.795 with a root mean squared error (RMSE) = 0.351, and *p*-value <0.0001. A summary of the model can be found in Table 2.

**Table 2:** Description of kinematic stepwise linear regression model.

Feature	Estimate	SE	t Statistic	*p*-value
(Intercept)	7.6141	0.13203	57.6	<0.0001
PC 1	−0.0002	<0.0001	−4.23	0.0002
PC 3	−0.0001	−2.6740	−2.67	0.0119
PC 9	−0.0003	−2.9866	−2.99	0.0055
PC 11	−0.0003	−2.9122	−2.92	0.0066
PC 12	0.0002	0.0001	1.83	0.0772
PC 13	0.0003	0.0001	2.64	0.0130
PC 16	0.0007	0.0002	4.73	<0.0001
Sex	0.3877	0.16523	2.35	0.02551


(7) Y=PC1+PC3+PC9+PC11+PC12+PC13+PC16+SEX

### Principal component analysis and linear regression (electromyography)

In the assessment of the sEMG data, the first 33 PCs were retained, which explained a cumulative 95.7% of the variance in the sEMG data set (Figure 5B). Individually, these PCs displayed a weak-moderate linear correlation with sprint velocity (*R* = 0.01–0.33), as with the kinematic data, this suggested that there are multiple neuromuscular factors that influence peak sprint velocity. Stepwise linear regression revealed that PC 1 (*p* = 0.011), PC 5 (*p* = 0.019), PC 21 (*p* = 0.016), PC 22 (*p* = 0.105) and sex (*p* = 0.0001) were significantly associated with the dependent variable, which was maximal horizontal sprint velocity. The linear regression model displayed an *R*^2 ^= 0.586 with a RMSE = 0.444, *p*-value = 1.64e-05. A summary of this model is presented in Table 3.

**Table 3:** Description of sEMG stepwise linear regression model.

Feature	Estimate	SE	t Statistic	*p-*value
(Intercept)	7.306	0.154	47.306	<0.0001
PC 1	−0.0008	0.0002	−3.105	0.0107
PC 5	−0.001	0.0004	−2.873	0.0189
PC 21	−0.0018	0.0007	−2.535	0.016
PC 22	0.002	0.0008	2.709	0.105
Sex	0.703	0.162	4.342	0.0001


(8) Y=PC1+PC5+PC21+PC22+SEX

### Functional interpretation of kinematic PCs

A summary of the biomechanical meaning of each individual PC, as obtained *via* SCR, can be found in Table 4, and a detailed analysis of each PC is presented in the Supplementary Figures (S2–S8). MCR was completed using PC 1, 3, 9, 11, 12 and 15, together these PCs represent 42.7% of the total variance in our data set.

**Table 4:** Summary of explained variance and biomechanical interpretation for PCs retained from the kinematic dataset.

PC	Explained Variance (%)	Biomechanical Interpretation
1	**25**.**2**	Timing and sequencing of the contralateral upper body mechanics relative to the lower body mechanics
3	**10**.**3**	Height of heel recovery Horizontal acceleration of head Range of motion of the elbow, shoulder, and spine
9	**2**.**7**	Transverse plane asymmetry of the lower limb during the swing phase and horizontal head acceleration
11	**1**.**7**	Trunk inclination across the entire gait cycle
12	**1**.**7**	Transverse plane asymmetry of the lower limb during the stance and early swing phase and dynamic trunk extension
13	**1**.**7**	Trunk inclination and frontal plane shoulder range of motion
16	**1**.**1**	Mid stance heel width

**Figure 6:**
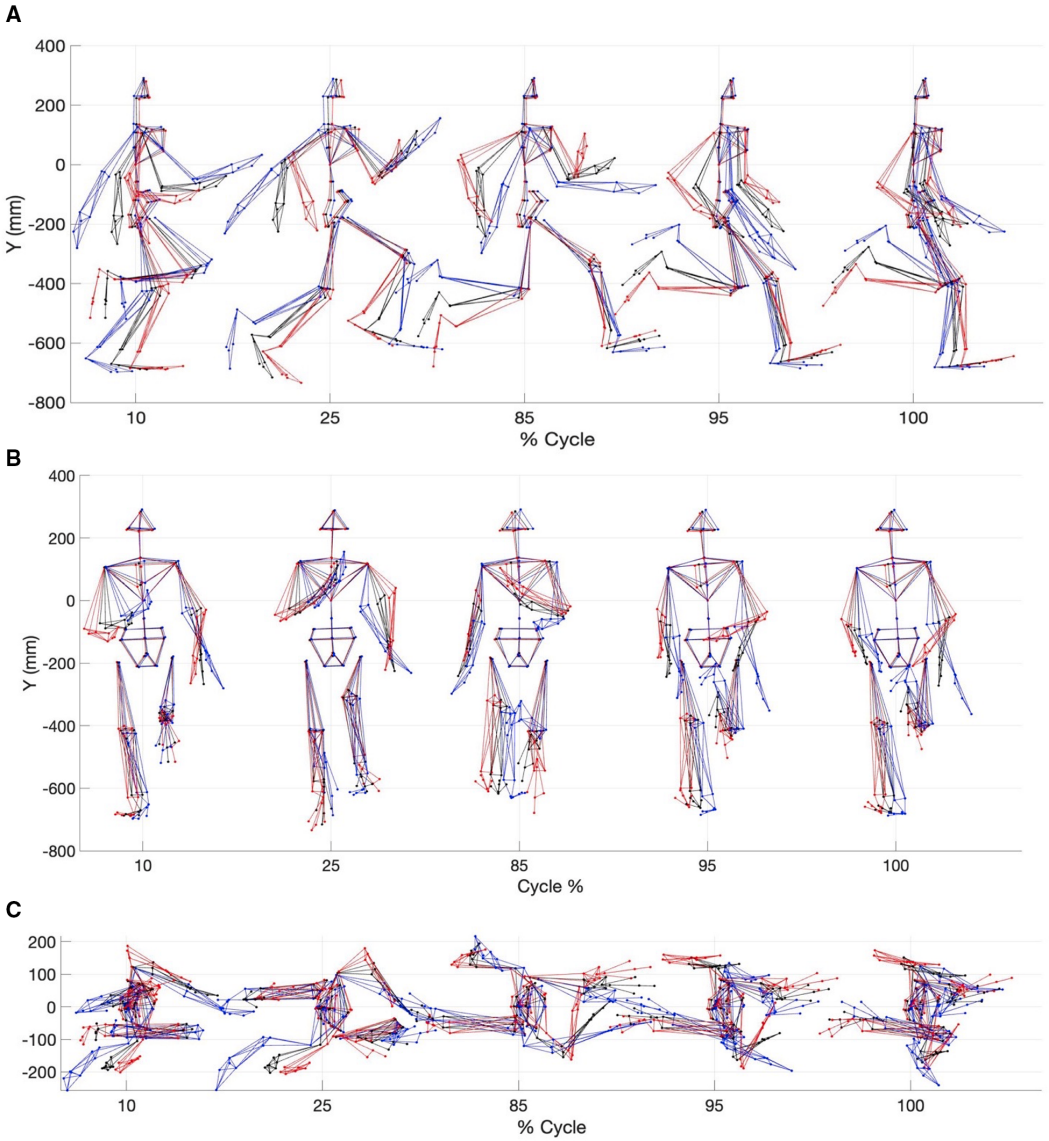
Multi-component reconstruction of PCs 1, 3, 9, 11, 12 and 6 derived from kinematic data. (**A**) Sagittal plane view; (**B**) Frontal plane view and (**C**) Transverse plane view. The red avatar represents the 5th percentile (slow), black represents the mean and blue represents the 95th percentile (fast).

Reconstructed motion data using MCR allowed visualization of the differences between a slow sprint velocity and a fast sprint velocity. For example, during the gait cycle fast sprint velocity MCR displayed better coordination between the upper and lower body (Figure 6). Specifically, this was noted with a more in-phase contralateral arm and leg swing during touch down and toe off, a dynamic trunk extension at toe off. Additionally, the fast sprint velocity MCR revealed a faster knee drive, higher heel recovery, asymmetrical leg swing, less horizontal head acceleration and an earlier onset of thoracic rotation, amongst others (Figures 6A–C).

### Interpretation of electromyography PCs

**Figure 7:**
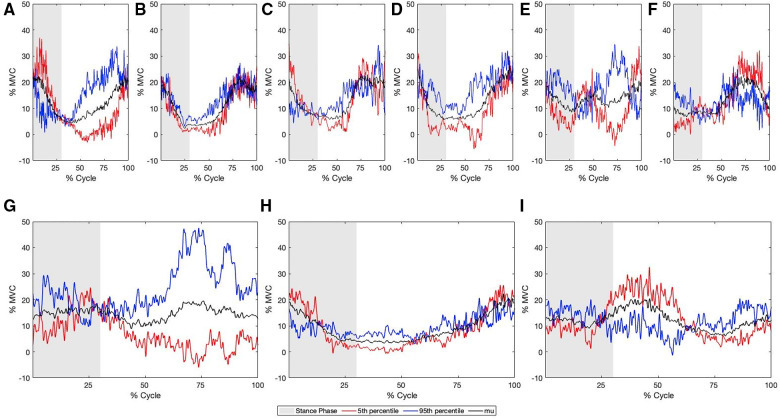
Multi-component reconstruction for PCs 1, 5, 21 and 22 derived from the sEMG for (**A**) GAS, (**B**) BF, (**C**) GMAX, (**D**) GMED, (**E**) LES, (**F**) LD, (**G**) EO, (**H**) VLO, (**I**) RF. Blue represents the 95th percentile sprinter (fast), Red represents the 5th percentile sprinter (slow), and grey represents the stance phase.

A summary of the biomechanical meaning of each PC can be seen in Table 5, and a detailed description is presented in the Supplementary Figures (S9–S12). MCR was completed using PC 1, 5, 21 and 22 together these PCs represented 21.9% of the total variance in our data set. MCR revelated that PCs scaled the GAS, GMAX, LD, EO, VLO and RF as a difference feature (Figure 7).

**Table 5:** Summary of explained variance and biomechanical interpretation for PCs retained from the EMG dataset.

PC	Explained Variance (%)	Biomechanical Interpretation
1	**13**.**1**	Phase shift feature for the musculature of the posterior chain Magnitude scaler for EO Difference feature for VLO and RF
5	**5**.**9%**	Difference features for the GAS, GMED, LD, EO, VLO, RF and GMAX
21	**1**.**5%**	No biomechanical meaning
22	**1**.**4%**	No biomechanical meaning

Reconstructed sEMG waveforms using MCR allowed for the visualization of the difference between slow and fast sprint velocities. Faster sprint velocities were associated with greater activation of the BF earlier in the gait cycle, an earlier peak magnitude of the LES, greater of the GAS prior to touchdown, greater activation of the GMAX during the swing phase and less during the stance phase, greater activation of the LD during the stance phase and less during the swing phase, greater EO activation throughout the entire gait cycle, less VLO activation during the stance phase and greater activation during the swing phase, less activation of the RF during the early swing phase, greater GMED activation during the late stance and swing phase (Figure 7).

## Discussion

This study aimed to identify key neuromuscular and kinematic determinants of peak horizontal sprint velocity in a large group of university-aged athletes. After collecting full-body kinematics and nine channels of sEMG on a large heterogeneous group (*n* = 40) of sprint-based athletes, PCA was used as a data reduction strategy for the kinematic and sEMG data sets separately. The retained PCs, biological sex, height, and age were used as independent variables into stepwise linear regression to find a correlation with peak sprint velocity. Significant multivariate regression models were generated for both kinematic (*R*^2 ^= 0.795) and sEMG (*R*^2 ^= 0.586) features identified using the retained PCs and biological sex.

### Sprinting kinematics

The features derived from the kinematic data set outperformed those from sEMG data set in the association with peak sprint velocity, as noted by a higher *R*^2^ value in the multivariate regression derived from kinematic features. This discrepancy may exist due to a number of complimentary factors. (1) The kinematic data set consists of fewer PCs to explain >95% of the variance in the data set, compared to the sEMG data set. It is possible that this is linked with the stochastic nature of sEMG signal. (2) The kinematic data set consists of a more complete data set, as it included the time varying position of 64 markers, which defined the position of all joints and segments in the body. While in contrast, the sEMG data set consisted of the time-varying activation patterns of only 9 muscles, spanning across the contralateral lower body and upper body. (3) Dynamic sEMG has several limitations due to the biophysics of the signal. For instance, the sensor is attached to a muscle, which displays viscoelastic properties, which can result in a non-uniform shape throughout a dynamic movement such as sprinting. This means the signal is susceptible to inconsistent pick-up volume and motion artifact, which results in physiologically irrelevant variance and can reduce the predictive power of a data set. (4) The human movement system is a complex system that is constructed through the interaction of many different subsystems, sEMG provides information on the neuromuscular activity of a muscle, however this is only one subsystem that may influence sprint performance. Specifically, sEMG signals do not capture any potential contributions of passive tissues (i.e., thoracolumbar fascia) which may affect the performance of a sprinter. In contrast, kinematic data represents human movement on a macroscopic level, which allows for the behaviour of all-sub-systems (i.e., passive, active, neural) to be analyzed. This may allow for kinematic data to be a better predictor of sprint velocity, since the data collected are a more holistic evaluation of the behaviour of the human movement system.

To improve sprint performance, an athlete must optimize the coordination of their entire body, to achieve peak horizontal sprint velocity. To do this the sprinter must absorb and then produce force during the stance phase. An effective transition between the two phases of the stance phase minimizes time on the ground and maximizes forward acceleration of the centre of mass. During the early stance phase, it has been shown that faster sprinters produce a larger amount of vertical ground reaction force. Previously, it has been shown the increased body mass (11) and hip angular velocity (23) appear to aid in the generation of vertical ground reaction force. Further, another important characteristic of increased force production may be the total sum of downward acceleration represented at the center of mass. Thus, it is possible that the timing of the contralateral arm and leg swing may also impact the total amount of vertical ground reaction force during the early stance phase, as it is possible that by optimizing the relationship between the upper and lower body there could be a force summation effect that is driven by the additional acceleration of the upper body. In reviewing the kinematic features, PC 1 and PC 3 demonstrated differences in the timing and magnitude of the arm swing. PC 1 represented differences in the timing of the arm swing, relative to the lower leg swing, meanwhile PC 3 represented differences in the timing of the arm swing. These differences also exist in the MCR which demonstrated a greater in-phase coordination between the contralateral arm and leg swing. Specifically, during tough down it can be seen that as the foot attacks the ground, the contralateral arm completes a downward motion that better aligns with foot strike in faster sprinters. It is possible that this may have a summative effect on the ground reaction force characteristics as the acceleration of the arm swing can be utilized to create a high ground reaction force, and in turn sprint velocity.

Vertical take-off velocity at toe-off has been shown to be associated with sprint velocity, through an increase in stride length (7). Similar to the early stance phase coordination between the upper and lower body, a similar effect was observed at toe-off in the visual appraisal of the MCR. During toe-off it was observed that as the stance side leg pushes off the ground, the contralateral arm swings upwards to aide in the projection of the centre of mass. This may also have a summative effect on centre of mass dynamics, where the acceleration generated from the upward arm swing can lead to an increase in vertical take-off velocity, increase stride length and thus improve sprint velocity. Although the relative timing of the contralateral arm and leg swing has not been studied in isolation, it has been shown that the arm swing can play an important role in the generation of greater vertical impulse (24), stride length (25) and horizontal velocity (25). Vertical take-off velocity may be further enhanced by the dynamic trunk extension we noticed in PCs 1, 3, 12 and our MCR. Specifically, we observed that the extension of the trunk occurred about toe-off, which suggests that this may aid in generating vertical take-off velocity. While this is the first study to our knowledge that has established a link between trunk coordination and sprint velocity, it is worth noting that some work has been done in this area and has shown positive correlations between a smaller inclination angle of the thorax and sprint velocity (4).

Ultimately, what goes up must come down, and in sprinting the forces associated with ground contact are estimated to be up to 5× the person's body weight (26). As a result, force attenuation is a key component in the skill of sprinting. Previously it has been shown that running can disrupt information to the vestibular and visual system (27) which can ultimately change an individual's full body coordinative strategy during running (28). Force attenuation during sprinting presents an interesting challenge to the CNS, as it is thought that knee flexion plays a large role in force attenuation during sub-maximal running (29). However, in sprinting knee flexion is minimized to facilitate increases in sprint velocity (7) which suggests that other joints and segments must be optimized to attenuate force. Interestingly, the data presented here corroborate some of these observations. As the movements associated with faster sprint velocities minimize the acceleration of the head by keeping it horizontal throughout the sprint. Interestingly, the horizontal tilting of the head on the slower MCR occurs asymmetrically, which also mirrors the asymmetry seen in the lower limb. Specifically, a more internally rotated trunk and pelvis and an arm swing away from the body was observed. It appears that these strategies may allow for the acceleration of the head to remain more constant, which has been previously reported as a compensatory strategy to attenuate force during running and walking (28, 30–32). While this is largely speculative at this point, these findings do warrant further investigation into whether these strategies are associated with the minimization of horizontal head acceleration.

The findings from our study suggest that a variety of key coordinative features are associated with improved sprint velocity. Specifically, the findings demonstrate that improved coordination between the upper and lower body may be related to the optimal force production strategies associated with improved sprint velocity (33, 26). Future work in this area should validate these assumptions by understanding the link between the contralateral arm and leg swing, and the dynamic trunk extension strategy demonstrated here and its impact into well-known biomechanical determinants of sprint performance. This includes determinants such as high vertical ground reaction forces during the early stance phase (26), direction of the ground reaction force (33), stride length (7) and stride rate (7).

### Sprinting electromyography

Although the predictive capacity of the sEMG data set was inferior to that observed from the kinematic data, several novel findings were observed. Specifically, PC 1 displayed a systematic phase shift feature in the posterior muscles that attach to the thoracolumbar fascia (Supplementary Figure S9). This systematic shift in the activation of the posterior musculature to later in the gait cycle may serve to maximize the acceleration of the lower leg during touch-down. Previous work by Clark and colleagues (2020) has shown that the angular kinematics of the thigh and ankle are closely related. This close relationship was proposed to be advantageous because the velocity gained from the hip was transferred to the shank at impact. This increase in foot velocity at touch down can be advantageous as it has been shown to create a larger vertical ground reaction force (23), which has been demonstrated to be differentiator between sprinters and non-sprinters (26). To our knowledge, this is the first study to evaluate the impact the muscles that attach to the thoracolumbar fascia have on sprint performance. These preliminary findings along with a strong theoretical foundation warrant further exploration into the role the coordination of these muscles have on sprint performance. Specifically, future studies should aim to understand the significance of this pattern by these muscles through muscle force modelling, synergist analysis, and co-contraction indices in addition to the SCR and MCR analyses implemented here.

To maximize the forward progression of the centre of mass during the sprint cycle the athlete must minimize braking forces, and simultaneously increase the propulsive forces. Previous work has shown that pre-activation of the hamstrings and plantar flexors increase with running velocity and may prevent unnecessary breaking forces during the contact phase of the sprint (34). While, both muscle groups are prone to injury, hamstring injuries have been a common cause for concern as the prevalence is high in both field based sports and track and field events (35). Recently, it was shown that a proximal neuromuscular control strategy may be associated with decreased occurrence of hamstring injuries in a large group of amateur soccer players (36). Specifically, greater activation of the gluteal group during the early swing phase and greater activation of the trunk muscular during the back swing phase was associated with a decreased prevalence of hamstring injuries in their data set. The activation patterns seen in this data set (37) mirror those seen in our data set. Where greater activation of the gluteal muscles is seen in the early swing phase, and greater activation of the trunk musculature is seen during the late swing phase. This may ultimately decrease the strain placed on the hamstring during late swing and early stance as a posteriorly oriented pelvis, would decrease the passive strain placed on the hamstring muscle group as the ischial tuberosity would be closer to the insertion point of the various muscles of the hamstring muscle group. This perhaps suggests that the neuromuscular strategy utilized to maximize sprint velocity may also help minimize the occurrence of injury. However, caution is advised when extrapolating functional interpretation from our sEMG dataset, as a limitation in this model is the reconstruction artifact that is seen in our MCR sEMG figures (Figure 7).

## Limitations

Although the approaches taken with this research have some fundamental strengths, they also have some limitations worth considering. The first limitation is the use of wearable technology which does not provide gold-standard kinematic data. Although validation studies have shown good agreement between IMUs and optical motion capture systems (37), the sensors used in this study are subject to factors such as ferromagnetic interference and drift. To accommodate errors derived from the use of wearable IMU sensors many technical steps were taken to correct our data (i.e., drift reduction); however, it is possible that these technical treatments of our data were insufficient, and instrumentation noise may still exist in our dataset. A second limitation may include the feature selection strategy used for this analysis. Specifically, we decided to retain many PCs, so that we could capture a variety of modes of variation that explained sprint performance. There is the possibility that by doing this we may have biased our stepwise linear regression towards PC features which represent biomechanically or physiologically irrelevant phenomena (i.e., noise). Future work in PCA should focus on more objective PC selection criteria based on heuristics and statistical based selection criteria to ensure greater certainty that the appropriate dimensionality of the data is selected. Finally, the current work presents a relatively modest sample size (40 participants, 13 female and 27 males). An aim for future work would be to leverage larger datasets to identify fundamental sprinting phenotypes using clustering algorithms which would likely segment the strategies taken by males and females.

## Conclusions

Sprint performance is multifactorial in nature and is, in part, dependent on fine-tuning of motor coordination. The results of this study demonstrate the utility of using a data-driven approach to identify key kinematic and sEMG features in a large dataset. The framework developed here is foundational to developing a fundamental understanding of which coordinative strategy is associated with improved performance with the hope it will ultimately bridge the gap between sport biomechanists and sport coaches. As we have now created an objective framework that can be used to inform sport coaches in the modification of sprint technique to ultimately improve a specific functional movement outcome (i.e., sprint velocity). The kinematic model demonstrates that improved coordination between the contralateral arm and leg swing, more stable horizontal gaze and a dynamic trunk extension strategy are associated with improved sprint velocity. While the sEMG model revealed greater pre-activation of the calves, hamstrings during the swing phase, greater activation of the gluteal muscles during the early swing and greater activation of the trunk musculature during the late swing phase is associate with improved sprint velocity. Together, these results demonstrate the importance of whole-body coordination during sprinting. As a result, coaches should integrate special exercises to enhance the athlete's whole-body coordination, while continuing to enhance previously reported factors associated with improved sprint performance. Collectively, the results of this study demonstrate the potential for machine learning to positively impact modern coaching practices.

## Data Availability

The raw data supporting the conclusions of this article will be made available by the authors, upon reasonable request.

## Supplementary material

SUPPLEMENTARY FIGURE S1

Example of Residual Analysis – P015, LES.

SUPPLEMENTARY FIGURE S2

SCR for PC 1 A) Sagittal plane view B) Frontal plane view C) Transverse plane view. The blue avatar represents the 5th percentile (slow), black represents the mean and red represents the 95th percentile (fast).

SUPPLEMENTARY FIGURE S3

SCR for PC 3 A) Sagittal plane view B) Frontal plane view C) Transverse plane view. The blue avatar represents the 5th percentile (slow), black represents the mean and red represents the 95th percentile (fast).

SUPPLEMENTARY FIGURE S4

SCR for PC 9 A) Sagittal plane view B) Frontal plane view C) Transverse plane view. The blue avatar represents the 5th percentile (slow), black represents the mean and red represents the 95th percentile (fast).

SUPPLEMENTARY FIGURE S5

SCR for PC 11 A) Sagittal plane view B) Frontal plane view C) Transverse plane view. The blue avatar represents the 5th percentile (slow), black represents the mean and red represents the 95th percentile (fast).

SUPPLEMENTARY FIGURE S6

SCR for PC 12 A) Sagittal plane view B) Frontal plane view C) Transverse plane view. The blue avatar represents the 5th percentile (slow), black represents the mean and red represents the 95th percentile (fast).

SUPPLEMENTARY FIGURE S7

SCR for PC 13 A) Sagittal plane view B) Frontal plane view C) Transverse plane view. The blue avatar represents the 5th percentile (slow), black represents the mean and red represents the 95th percentile (fast).

SUPPLEMENTARY FIGURE S8

SCR for PC 16 A) Sagittal plane view B) Frontal plane view C) Transverse plane view. The blue avatar represents the 5th percentile (slow), black represents the mean and red represents the 95th percentile (fast).

SUPPLEMENTARY FIGURE S9

SCR for PC 1 the blue line represents a muscle activation pattern associated with faster sprint velocities, a red line is associated with slower sprint velocities, and black represents the mean.

SUPPLEMENTARY FIGURE S10

SCR for PC 5 the blue line represents a muscle activation pattern associated with faster sprint velocities, a red line is associated with slower sprint velocities, and black represents the mean.

SUPPLEMENTARY FIGURE S11

SCR for PC 21 the blue line represents a muscle activation pattern associated with faster sprint velocities, a red line is associated with slower sprint velocities, and black represents the mean.

SUPPLEMENTARY FIGURE S12

SCR for PC 22 the blue line represents a muscle activation pattern associated with faster sprint velocities, a red line is associated with slower sprint velocities, and black represents the mean.

Supplementary Table S1

Summary of Residual Analysis Results.
