# Supplementary figures and images for: A need for speed: Objectively identifying full-body kinematic and neuromuscular features associated with faster sprint velocities
Source: Front Sports Act Living. 2023 Feb 3;4:1094163. doi: 10.3389/fspor.2022.1094163 (PMC9936194; doi:10.3389/fspor.2022.1094163)

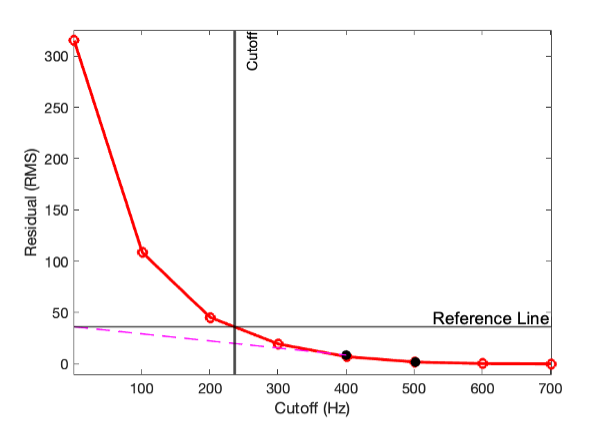

Supplement: Supplementary file 1 [file Image1.tiff]

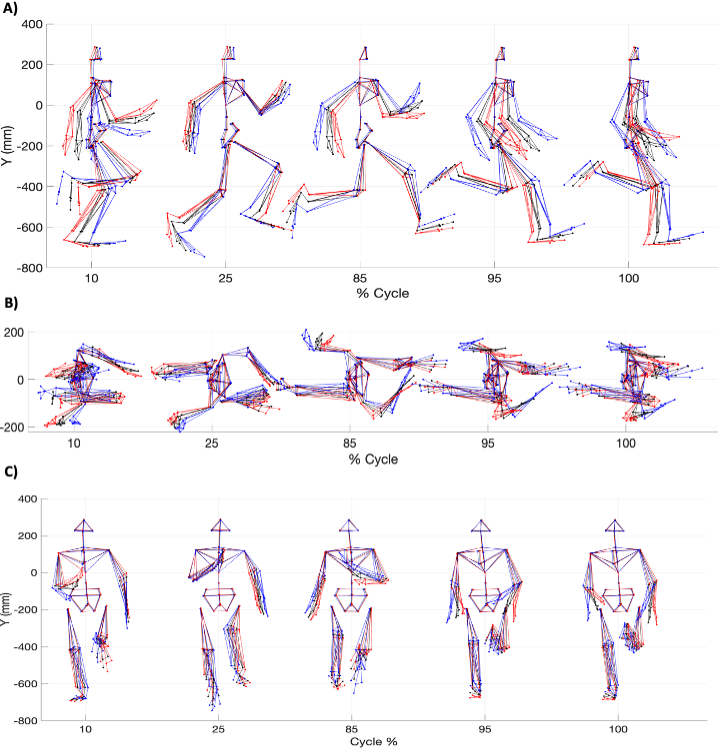

Supplement: Supplementary file 2 [file Image2.tiff]

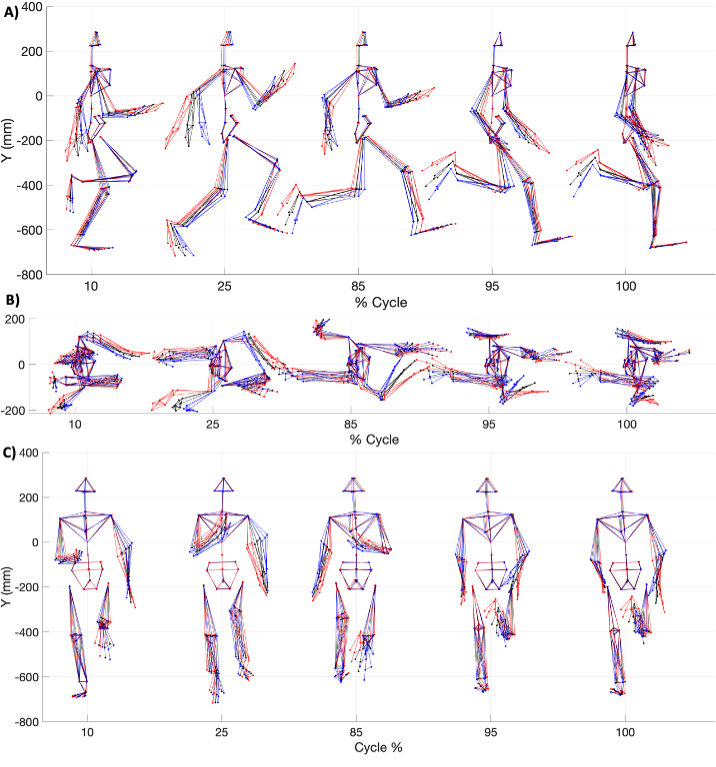

Supplement: Supplementary file 3 [file Image3.tiff]

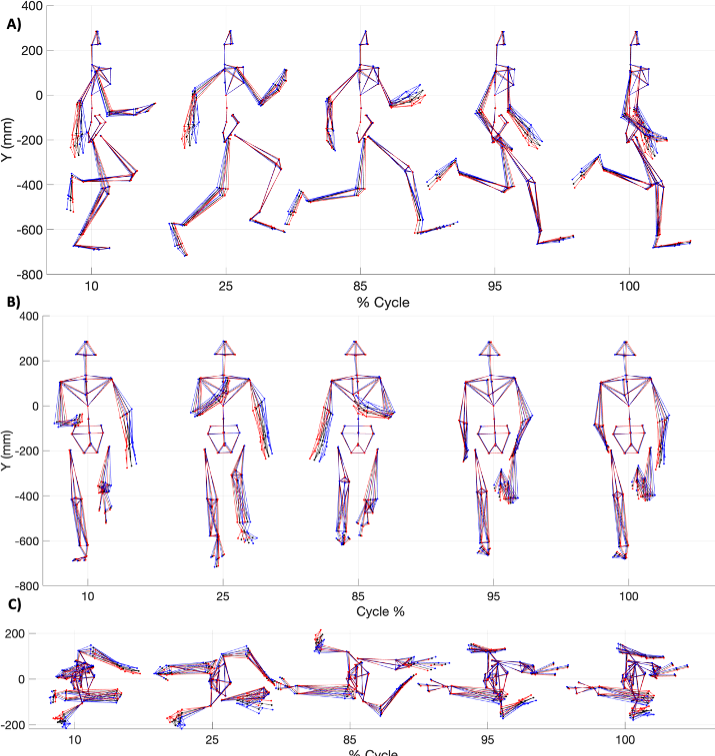

Supplement: Supplementary file 4 [file Image4.tiff]

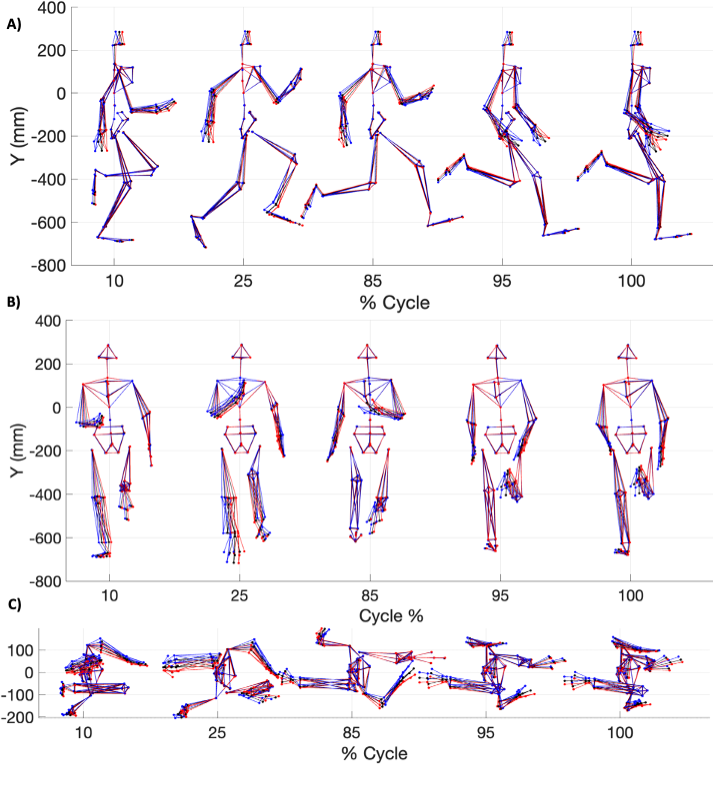

Supplement: Supplementary file 5 [file Image5.tiff]

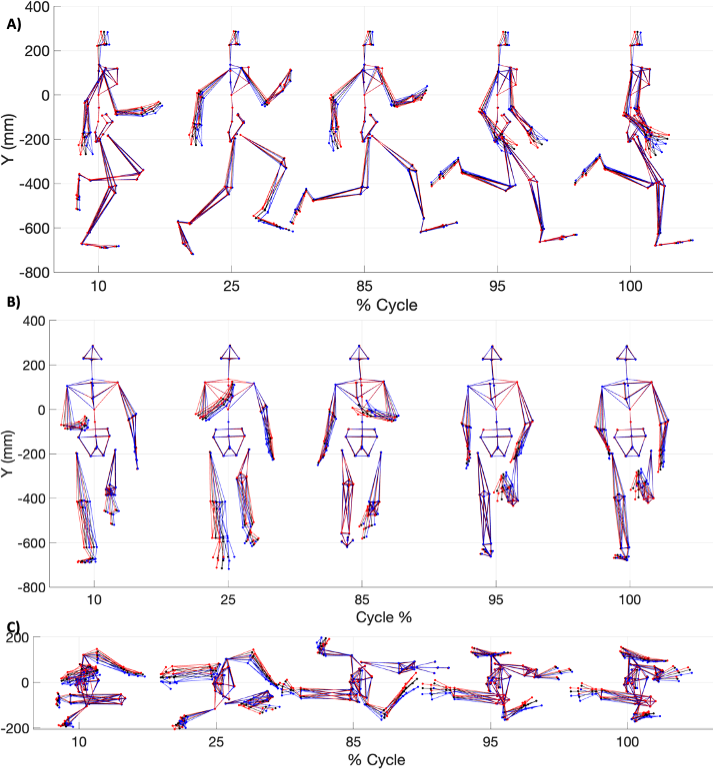

Supplement: Supplementary file 6 [file Image6.tiff]

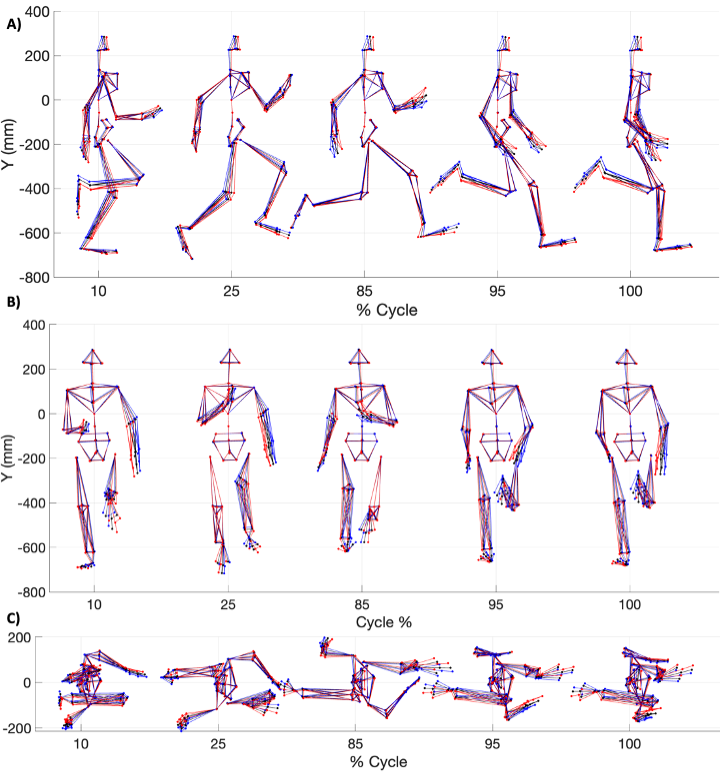

Supplement: Supplementary file 7 [file Image7.tiff]

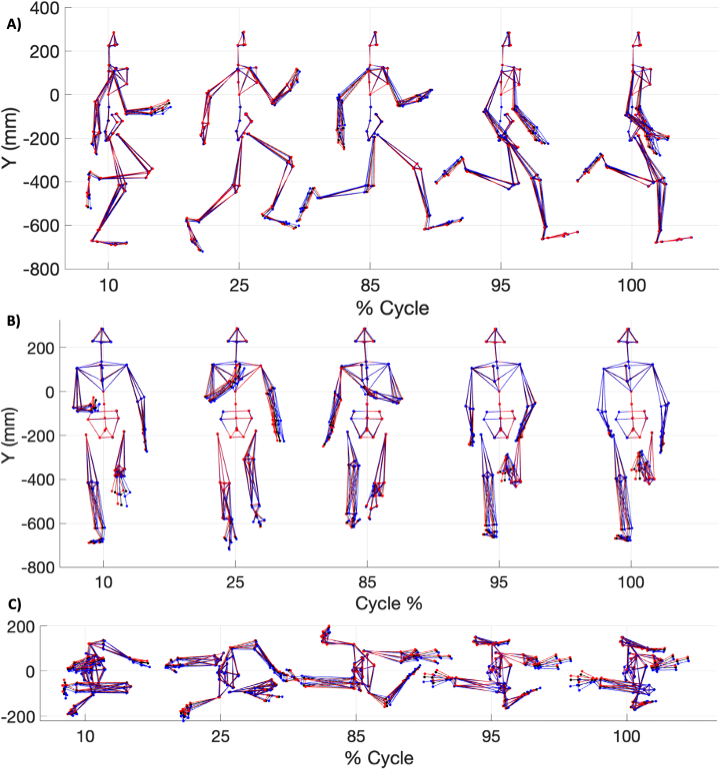

Supplement: Supplementary file 8 [file Image8.tiff]

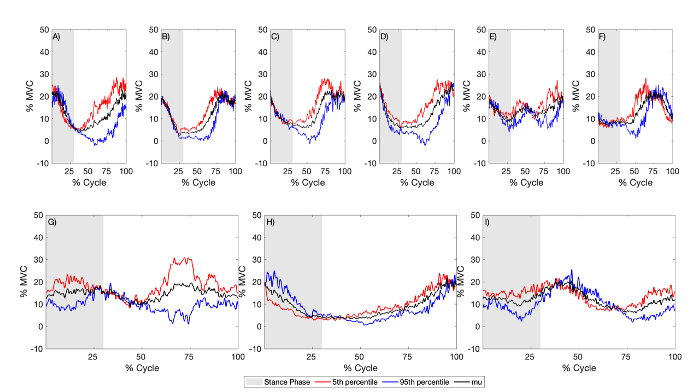

Supplement: Supplementary file 9 [file Image9.tiff]

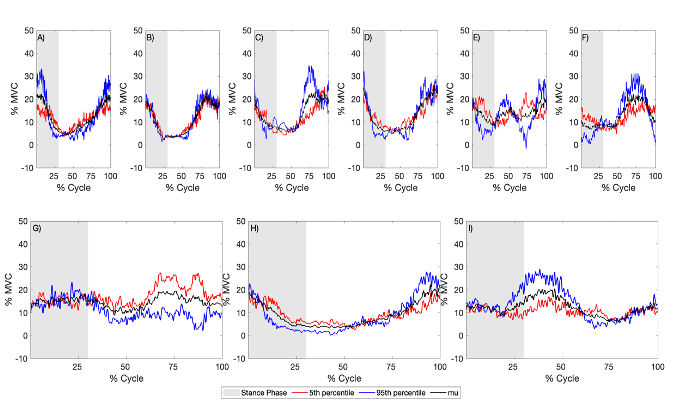

Supplement: Supplementary file 10 [file Image10.tiff]

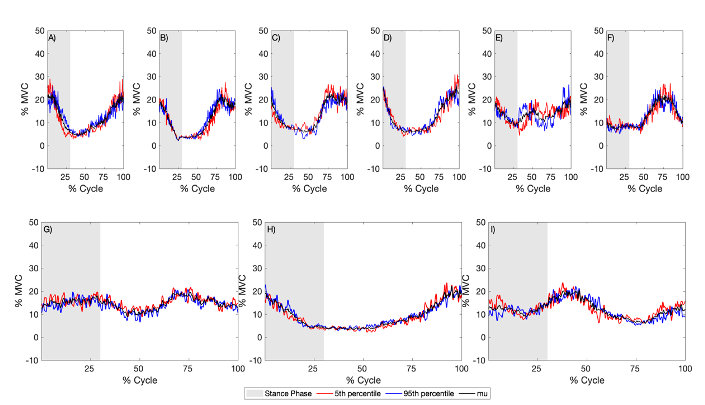

Supplement: Supplementary file 11 [file Image11.tiff]

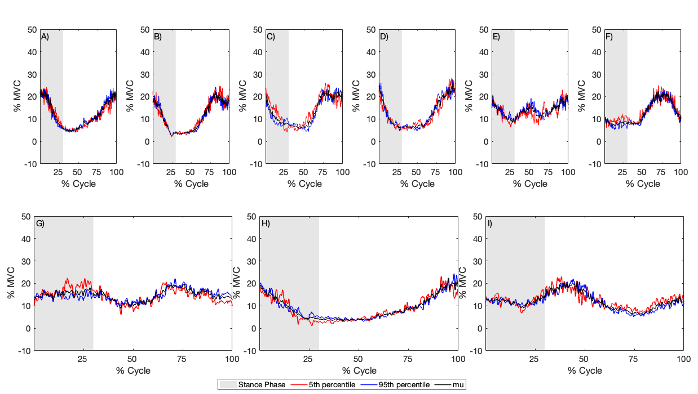

Supplement: Supplementary file 12 [file Image12.tiff]
