# Supplementary material for: A need for speed: Objectively identifying full-body kinematic and neuromuscular features associated with faster sprint velocities
Source: Front Sports Act Living. 2023 Feb 3;4:1094163. doi: 10.3389/fspor.2022.1094163 (PMC9936194; doi:10.3389/fspor.2022.1094163)
Supplement: Supplementary file 13 [file Table1.pdf]

Supplementary Material

Supplementary Tables:

Supplementary Table 1. Summary of Residual Analysis Results

Participant	Trial	VLO (Hz)	LUM (Hz)	OBL (Hz)	MED (Hz)	BIC (Hz)	MAX (Hz)	REC (Hz)	LAT (Hz)	GAS (Hz)	MCUTOFF (Hz)
**P001**	1	196	184	255	213	403	180	241	297	285	**251**
**P004**	3	203	178	265	222	354	169	188	190	313	**231**
**P005**	1	201	282	220	282	364	203	271	434	282	**282**
**P006**	1	286	229	292	203	296	197	214	243	349	**257**
**P019**	2	273	192	301	254	235	235	271	248	341	**261**
**P015**	3	294	303	201	305	375	201	201	265	269	**268**

Figure Captions:

Supplementary Figure 1. Example of Residual Analysis – P015, LES

Supplementary Figure 2. SCR for PC 1 **A**) Sagittal plane view **B)** Frontal plane view **C**) Transverse plane view. The blue avatar represents the 5th percentile (slow), black represents the mean and red represents the 95th percentile (fast)

Supplementary Figure 3. SCR for PC 3 **A**) Sagittal plane view **B)** Frontal plane view **C**) Transverse plane view. The blue avatar represents the 5th percentile (slow), black represents the mean and red represents the 95th percentile (fast)

Supplementary Figure 4. SCR for PC 9 **A**) Sagittal plane view **B)** Frontal plane view **C**) Transverse plane view. The blue avatar represents the 5th percentile (slow), black represents the mean and red represents the 95th percentile (fast)

Supplementary Figure 5. SCR for PC 11 **A**) Sagittal plane view **B)** Frontal plane view **C**) Transverse plane view. The blue avatar represents the 5th percentile (slow), black represents the mean and red represents the 95th percentile (fast)

Supplementary Figure 6. SCR for PC 12 **A**) Sagittal plane view **B)** Frontal plane view **C**) Transverse plane view. The blue avatar represents the 5th percentile (slow), black represents the mean and red represents the 95th percentile (fast)

Supplementary Figure 7. SCR for PC 13 **A**) Sagittal plane view **B)** Frontal plane view **C**) Transverse plane view. The blue avatar represents the 5th percentile (slow), black represents the mean and red represents the 95th percentile (fast)

Supplementary Figure 8 SCR for PC 16 **A**) Sagittal plane view **B)** Frontal plane view **C**) Transverse plane view. The blue avatar represents the 5th percentile (slow), black represents the mean and red represents the 95th percentile (fast)

Supplementary Figure 9. SCR for PC 1 the blue line represents a muscle activation pattern associated with faster sprint velocities, a red line is associated with slower sprint velocities, and black represents the mean

Supplementary Figure 10. SCR for PC 5 the blue line represents a muscle activation pattern associated with faster sprint velocities, a red line is associated with slower sprint velocities, and black represents the mean

Supplementary Figure 11. SCR for PC 21 the blue line represents a muscle activation pattern associated with faster sprint velocities, a red line is associated with slower sprint velocities, and black represents the mean

Supplementary Figure 12. SCR for PC 22 the blue line represents a muscle activation pattern associated with faster sprint velocities, a red line is associated with slower sprint velocities, and black represents the mean
